# Comparative Evaluation of Induction Furnace Steel Rebars: Mechanical Properties, Microstructure, and High-Temperature Corrosion Behavior in Seawater

**DOI:** 10.3390/ma19112350

**Published:** 2026-06-02

**Authors:** Azmat Chandio, Iftikhar Ahmed Channa, Ayaz Ali Shah, Tasneem Pervez, Waqas Ahmed, Sarmad Feroze, Muhammad Ali Shar, Abdulaziz Alhazaa, Ali Dad Chandio

**Affiliations:** 1 Department of Metallurgical Engineering, NED University of Engineering & Technology, Karachi 75270, Pakistan; eng.azmat08@gmail.com; 2 Department of Energy and Environment Engineering, Dawood University of Engineering & Technology, Karachi 74800, Pakistan; aas@duet.edu.pk; 3 Department of Mechanical and Industrial Engineering, Sultan Qaboos University, Muscat 123, Oman; tasneem@squ.edu.om; 4 Department of Industrial and Manufacturing, NED University of Engineering & Technology, Karachi 75270, Pakistan; ahmed.22waqas@gmail.com; 5 School of Materials Science and Engineering (MSE), Nanyang Technological University (NTU), 50 Nanyang Ave, Singapore 639798, Singapore; sarmad.feroze@ntu.edu.sg; 6 King Abdullah Institute for Nanotechnology, King Saud University, Riyadh 11451, Saudi Arabia; mashar@ksu.edu.sa; 7 Department of Physics and Astronomy, King Saud University, Riyadh 11451, Saudi Arabia; aalhazaa@ksu.edu.sa

**Keywords:** steel, mechanical properties, induction furnace, microstructure, corrosion

## Abstract

The present investigation provides a comparative quality evaluation of Grade-60 steel rebars produced by induction furnace (IF)-based industries. In addition to analyzing quality variations in different industrial processes, this study aims to evaluate their compliance with international standards such as ASTM A615/615M. Samples of rebars from six local producers were collected, and their chemical compositions, microstructural features, hardness profiles, and tensile properties, including yield and tensile strengths, were analyzed. A special focus was given to analyzing the case–core microstructure, the presence of martensite rings, and impurity density, which substantially affect strength and ductility. Additionally, the electrochemical investigation was conducted at different temperature ranges, from 50 °C to 100 °C. Potentiodynamic polarization (PDP), electrochemical impedance spectroscopy (EIS) with open-circuit potential (OCP) was used to analyze the corrosion behavior of different samples in seawater. The finding suggests that the majority of rebars achieved the minimum requirements of ASTM 615 for Grade-60, except for some manufacturers, where minor deviation was noticed. However, significant variation in mechanical properties and chemical composition was observed among all six industries (A–F). Manufacturers are consistently exhibiting the highest mechanical properties, with a maximum yield strength of 580 MPa and tensile strength of 760 MPa (12 mm diameter sample). In contrast, various samples from E and F manufacturers recorded the lowest values, such as a minimum yield strength of 365 MPa (Industry F, 16 mm diameter) and a critically low elongation of 9.5% for sample E of 16 mm diameter. Microstructural analysis further confirmed the formation of martensite rim structures in samples of manufacturer A, evidencing the efficient thermomechanical treatment system installation in industries.

## 1. Introduction

Reinforcing steel bars (rebars) are the backbone of construction and infrastructure development all over the world. The steel industry has grown rapidly; approximately 50% of world steel usage is associated with rebars and other steel components utilized in construction [1,2,3]. Their role in providing strength, ductility, and structural integrity to concrete makes them a critical component in large projects. Among various grades, G-60 (420 MPa) rebars specified under standard ASTM A615 are widely used from residential to large industrial structures due to high yield strength, good weldability, and other properties [4]. The performance and reliability of these rebars, however, depend significantly on their chemical composition, manufacturing process, and quality control during production. Over the past few years, the demand for rebars in Pakistan has increased significantly, with long-steel production (including rebars) in the country increasing by nearly 33% in the fiscal year 2022 to approximately 6.36 million metric tons, with national rebar consumption estimated at 6.5–7.0 million tons per year [5].

In Pakistan, according to Pakistan Steel Melters and independent bodies like Pakistan Credit Rating Agency (PACRA) and Sustainable Development Policy Institute (SDPI), a significant share of rebar production is undertaken by the private sector, using scrap-fed induction furnace (IF) technology, followed by continuous casting, and thermomechanical treatment (TMT) through hot rolling and quenching [6]. This route is cost-effective and widely adopted because it allows rapid recycling of ferrous scrap into construction-grade steel [7]. However, due to differences in scrap quality, process parameters, and plant-level quality assurance practices, the final mechanical and microstructural properties of rebars often vary from one manufacturer to another, even though the production route is nominally the same [8]. This inconsistency is largely governed by the phase transformation kinetics during the rapid cooling stages of production. As investigated by Bharadwaj et al. (2022), the interplay between finishing temperatures and subsequent cooling rates dictates the final volume fractions of the constituent phases [9]. Maintaining consistent quality of rebars across different manufacturers is still a significant challenge. Strength, ductility, and corrosion resistance can be influenced by variation in chemical composition, including carbon, manganese, silicon, sulfur, and phosphorus [10,11]. Likewise, controlling the thermomechanical treatment process is vital in determining the depth of martensite rim and ferrite–pearlite core, which directly affects the strength of rebar. From an industrial standpoint, examining the quality of rebars from different producers is critical to identify process control flaws and ensure adherence to standards like ASTM A615 and BS 4449 [12]. It supports process improvements, increases market competitiveness, and responds to the growing demand for rebar.

Several studies worldwide have reported the influence of production route and heat treatment on the mechanical and metallurgical properties, including corrosion/rusting of reinforced bars [13,14,15]. However, limited work has been carried out on a systematic, industrial-linked examination of locally produced steel rebars. Most of the available data focus on laboratory-scale analysis or single plant observations, leaving a gap in understanding of overall process consistency and product uniformity across different producers operating under similar routes. For instance, Abro et al. 2017 [16], examined the influence of intercritical heat treatment on scrap-based rebars and reported that the process produced a dual-phase structure that enhanced strength and ductility, highlighting the benefits of controlled thermomechanical processing. Similarly, a comparative investigation between blast furnace and induction furnace produced rebars. The results indicated that induction furnace rebars generally contained higher levels of alloying elements and exhibited a thinner martensitic rim compared to blast furnace products. These differences affected the tensile-to-yield strength ratio and overall ductility, underscoring the importance of process control and chemical composition management in IF-based production [17]. Tadele et al. (2023) [18] conducted a large-scale analysis of North American rebars. Most of the rebars complied with ASTM A615 standards; a noteworthy fraction failed to meet mechanical property requirements. Rafi et al. (2014) surveyed Pakistani rebars and found up to 60% failed elongation criteria despite acceptable chemistry, reinforcing concerns about ductile–brittle transition risks [19]. In Karela, Varghese et al. (2024) [20] conducted optical microscopy on site-collected rebars. Samples revealed inconsistent martensite formation and poor-quality assurance practices at the plant level. Their work highlights the importance of enforcing standardized microstructural testing before deployment in construction projects. In a research investigation, a comparison between stainless steel, plain, and TMT rebars under mechanical and corrosion testing using Tafel plots and hardness profiling was conducted. The classic U-shaped hardness profile in TMT bars (outer martensite, inner ferrite–pearlite) was identified, and confirmed that stainless rebars significantly outperformed plain and TMT bars in both strength and corrosion resistance, particularly in aggressive environments like HCl and seawater [21].

Collectively, these studies underline the critical role of process control, alloying, and quality assurance in ensuring rebar reliability. They also reveal gaps in uniformity across manufacturers, even when similar production routes are used, making systematic industrial-scale evaluations essential for improving standards and structural safety. Furthermore, mechanical properties have been assessed in numerous studies, but there is still a lack of a comprehensive understanding of how these processing parameters and microstructural changes influence the corrosion resistance of rebars, particularly under aggressive environments and high temperatures. Previous studies have explored the effect of different processing routes on mechanical properties and low-temperature corrosion behavior; however, rather than optimizing a single thermal process under isolated laboratory conditions using a uniform starting material, this study focuses on a comparative industrial benchmarking analysis of commercially available products. Because different manufacturers utilize distinct raw materials and production technologies, evaluating these steels as-received introduces inherent industrial variables. Investigating these variations is essential for reflecting the actual market landscape, as it allows for a pragmatic assessment of how diverse commercial manufacturing practices influence the final physical uniformity and anticorrosion performance. To bridge this gap, this work conducts a detailed comparative investigation of locally manufactured steel rebars from six major induction furnace (IF)-based producers in the region. Samples from these selected industries, following a similar process chain, were collected to provide an established framework for comparison. The core focus is placed on evaluating fundamental quality parameters, including chemical composition, mechanical properties, and microstructures, and correlating them with corrosion behavior at different temperatures via open-circuit potential (OCP) and potentiodynamic polarization curves.

## 2. Material & Methodology

### 2.1. Sample Collections

A representative comparison of regional industrial manufacturing reliability was established by collecting Grade-60 rebars with different diameters (i.e., 12 mm, 16 mm, 20 mm) from six local manufacturers in Karachi city of Sindh Province, Pakistan. Manufacturers were designated as A, B, C, D, E, and F to provide precise traceability. In this study, several batches were examined from all the mentioned industries to make sure of repeatability in results. In order to guarantee experimental reproducibility and account for production line variability, three distinct manufacturing batches were sampled from each producer.

### 2.2. Chemical Composition

Optical emission spectroscopy (OES) was utilized to determine the chemical composition of all received samples. The primary alloying and residual elements, including C, Si, Mn, P, S, Cu, Ni, and Cr, were examined. Carbon equivalent (CE) was also determined using Equation (1).

(1) $$CE=C+\frac{Mn}{6}+\frac{Cr+Mo+V}{5}\frac{Ni+Cu}{15}$$

### 2.3. Mechanical Testing

Mechanical properties were evaluated using tensile and hardness tests. A tensile test was conducted using a rebar sample of 18 inches on a universal tensile machine (model SHT4106, supplied by SANS Testing Machine Co., Shenzhen, Guangdong, China). Total elongation was determined by utilizing a gauge length of 200 mm on each specimen. The tests were executed at a constant crosshead displacement rate of 5 mm/min to maintain uniform, quasi-static loading conditions. The cross-sectional hardness profile of the 20 mm rebars was mapped from surface to core across 7 different positions spaced at 1.5 mm intervals. At each position, three Vickers microhardness indentations were performed using Wolpert hardness tester (model 450SVA, from Wolpert Group, Ludwigshafen, Germany) and automatically converted to the Rockwell C (HRC) scale by the system’s software for direct industrial comparison.

### 2.4. Microstructural Examination

Microstructural analysis was used to determine the relationship between phase distribution and mechanical performance. Metallographic specimens were sectioned, ground using progressively finer silicon carbide emery papers (from 200 to 1600 grit), and polished to a mirror finish utilizing a 1 μm alumina suspension on a cloth wheel. The etching was performed by 2%Nital that was prepared by using 2 vol.% analytical-grade nitric acid (HNO_3_ of 68% purity, supplied by Sigma-Aldrich, St. Louis, MO, USA) and 98 vol.% ethanol (C_2_H_5_OH, 99.9% purity from Merck, Rahway, NJ, USA). Etching was performed at room temperature for a duration of 10 s, followed immediately by an absolute ethanol rinse and air drying to prevent atmospheric staining before optical microscopic examination.

### 2.5. Corrosion Testing

A three-electrode electrochemical glass cell that was connected to the Reference 600TM electrochemical workstation (Gamry Instruments, Warminster, PA, USA) and control software (Gamry Framework, version 7.10) was used to conduct electrochemical measurements. The rebar samples with a 20 mm diameter were used as the working electrode (WE). The received samples were cleaned thoroughly, and the cut cross-sectional ends were insulated and sealed with a non-conductive epoxy resin. This preparation exposed a cylinder with 117 mm of surface area to interaction with the electrolyte. A platinum mesh was used as counter electrode (CE), and a saturated calomel electrode (SCE) was employed as reference electrode (RE). Steady-state open-circuit potential (OCP) conditions were established via a 30 min immersion period before initiating measurements. Potentiodynamic polarization tests were then carried out with a potential range of −0.4 V to +0.4 V, with a scan rate of 1 mV/s and three different temperatures (50, 75, and 100 °C). For the elevated temperature testing specifically 100 °C, the cell temperature was regulated within ±1 °C utilizing a circulating oil bath governed by a digital temperature controller and a submerged thermocouple. To counter the significant water evaporation expected at 100 °C, which would otherwise alter the NaCl concentration and artifactually skew the electrochemical potentials, the cell was equipped with a water-cooled reflux condenser system. This setup successfully condensed escaping vapors and recycled them into the bulk electrolyte, maintaining a constant solution concentration and volume throughout the duration of the electrochemical measurements. The corrosion current densities (I_corr_) and Tafel slopes, such as anodic (βa) and cathodic (βc), were derived by extrapolating the Tafel plots using EC-LAB software (version 11.33).

### 2.6. Electrolyte

Natural seawater collected directly from the Arabian Sea coast was used as the corrosive electrolyte medium to replicate coastal and marine conditions (Karachi, Pakistan). The chemical composition of the filtered electrolyte was systematically analyzed and verified to feature a baseline pH of 8.3, a total dissolved solids (TDS) content of 439 mg/L, 155 mg/L of chloride (Cl^−^) ions, and 53 mg/L of sodium (Na^+^) ions.

### 2.7. Industrial Processing Routes and Parameters

IF-based rebar production in Pakistan typically follows the same overall processing sequence. However, variation in parameters are caused due to technology and equipment capacity and operational practices among individual plants. In general, steel scrap is fed into the furnace, melted at a controlled temperature, and slag is separated before tapping into the ladle. Billets of 130 mm × 130 mm or 150 mm × 150 mm are cast using a continuous cast machine, and induction heating is carried out as per the required temperature above recrystallization. The rolling mill reduces the billet to the final bar diameter, achieving the target temperature for effective quenching.

In the TMT stage, bars experience controlled water quenching to form a hardened martensitic case, which is then self-tempered by the core heat. A cooling bed is used for final cooling to create a ferrite–pearlite core. Variations in temperature, water pressure, and cooling rate among vendors have a considerable impact on martensite rim thickness, bainitic transition zones, hardness gradients, and, ultimately, mechanical properties. Table 1 shows the parameters for 20 mm bars from various vendors.

## 3. Results and Analysis

### 3.1. Chemical Composition of Rebars

Table 2 represents the chemical composition of manufactured rebars from different industries (12 mm, 16 mm, and 20 mm), which was determined using the spectrometer model SPECTROMAXx (SPECTRO Analytical Instruments, Kleve, Germany). C and Mn were two major alloying elements that contribute most to the formation of martensitic structure and the strength of rebars, respectively. It can be observed that the C fraction is in the range of 0.20 to 0.265%, whereas Mn composition varied from 0.621 to 0.844%. Additionally, Si percent was also within limits (0.158–0.289%), offering improved fluidity of molten metal and smooth casting. The S and P can cause hot shortness by forming FeS, and cold shortness by segregating at grain boundaries, respectively. According to ASTM A615, the maximum allowed levels for sulfur and phosphorus are strictly limited to 0.06 wt.%. However, it was observed above 0.04 and 0.035%, respectively, i.e., relatively higher than EAF-produced rebars [22]. The carbon equivalent (CE) is also significant for weldability; higher CE values can increase the brittleness [23]. Higher CE could potentially increase the hardenability, thereby reducing the ductility. Consequently, it leads to the formation of hard, brittle microstructures (like martensite) and, thus, makes it susceptible to cracking, especially during processes involving rapid cooling, such as in welding. The range of CE was 0.0406 to 0.476, with the highest value seen in sample A of 12 mm.

### 3.2. Microstructural Characterization

**Figure 1:**
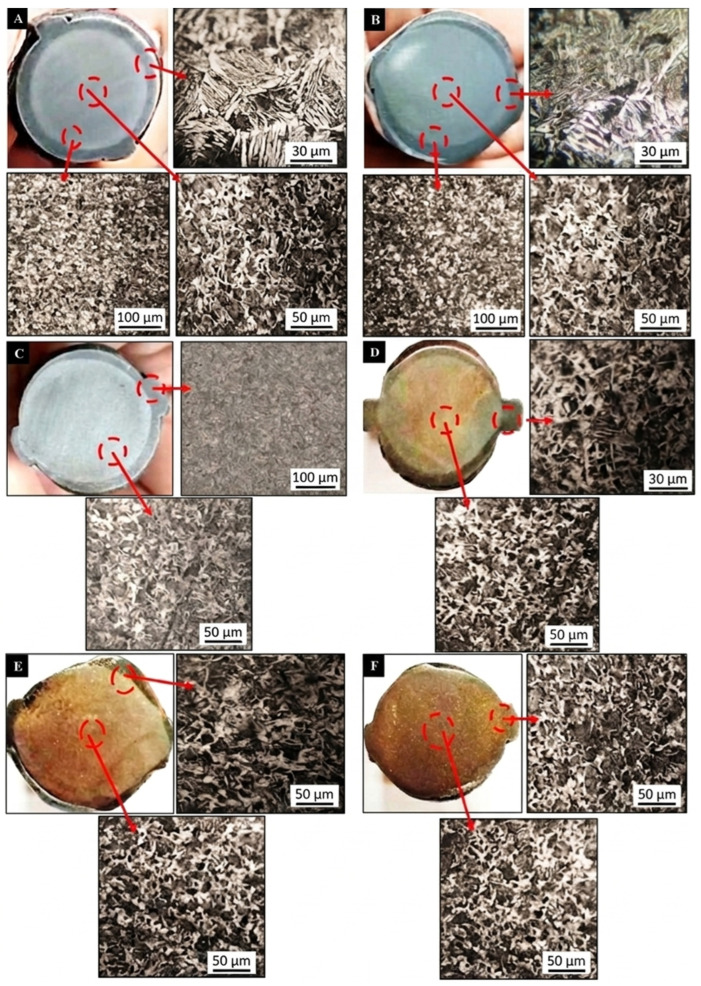
Macrostructural cross-sections and corresponding microstructural regions (case, core, and transition zones) for the investigated rebar manufacturers (**A**–**F**).

Different microstructural regions were present on the cross-sections of 20 mm diameter rebars under examination, as shown in Figure 1A–F. Significant variation in phase morphology and martensite rim continuity was noticed, typically influenced by variation including carbon content and alloying elements in scrap, heating temperature, quenching pressure, and cooling rate. These factors controlled rim thickness, martensite formation, and the presence of such phases.

Notably, the most desired microstructural features were found in samples A and B. Both samples developed a continuous and well-defined martensitic rim, formed by rapid quenching and a refined ferrite and pearlite core attributed with controlled self-tempering [24]. The rim comprises fine lath martensite, uniformly distributed around the circumference, which is critical because rim continuity determines how effectively the material can withstand high tensile strength and fatigue loads. Moreover, the refined pearlite colonies in the core promote strain uniformity during loading, enabling high elongation without premature necking. A bainitic transition zone between core and case was observed in samples A and B [21]. This intermediate phase acts to improve toughness and mitigate abrupt hardness changes.

Metallographic analysis using ImageJ software (Version 1.54p) to investigate optical micrographs across multiple fields of view confirmed that samples A and B have approximately 450 and 370 µm, respectively, with martensite forming over 90% of the rim structure. Bainite was present in minor amounts, while the core was made of refined ferrite and pearlite matrix. In contrast, samples of C manufacturers presented a marginally acceptable microstructural profile, characterized by a thinner rim consisting of tempered martensite, while the D sample showed lathe martensite on the surface with pearlite. Both C and D reached the same yield strength, despite microstructural differences. However, D displayed lower tensile strength and hardness, which may be a result of mixed phases.

The poorest microstructural integrity was observed in samples E and F, where martensitic rims were discontinuous or nearly absent. Martensite rims were discontinuous or almost absent, corresponding to the lowest carbon content and inadequate quenching pressure during processing. Among all, sample E showed serious flaws, with transformation products suggesting low functional strength with composition in a range.

At least five different fields of view were methodically assessed for each sample over three different production batches in order to guarantee the statistical reproducibility of these microstructural observations. This thorough analysis verified that the non-metallic inclusion distributions, case-to-core morphologies, and peripheral rim uniformity changes were consistent structural characteristics of each producer.

**Figure 2:**
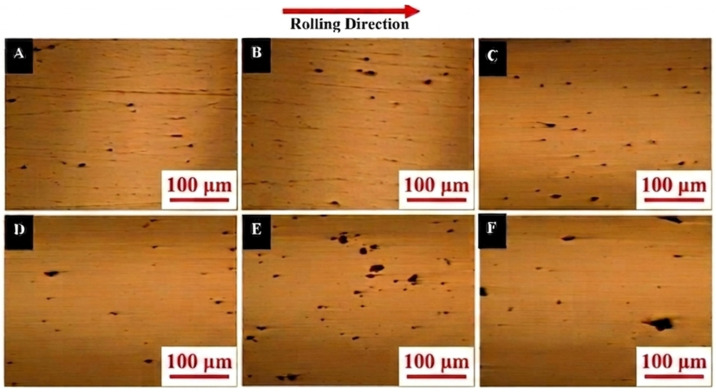
Optical micrographs displaying non-metallic inclusion distributions and morphologies along the rolling direction for the different rebar manufacturers. (**A**,**B**) Fine, highly elongated inclusions, (**C**,**D**) moderate inclusion density, and (**E**,**F**) coarse, clustered inclusions.

Figure 2 depicts the inclusions in the rolled 20 mm bar from each plant. Heterogeneity was observed in all samples, showing evidence of inconsistent refining of IF-based production [25]. Samples A and B displayed fine, elongated inclusions aligned along the rolling direction, which is characteristic of controlled rolling high-quality scrap. Such morphology suggests that non-metallic inclusions were plastically deformed during rolling, reducing their detrimental effect on mechanical properties [20,26]. Conversely, samples C and D exhibited irregularly distributed clusters with varied morphologies, implying higher impurity content and inadequate rolling parameters. This is consistent with reports in the literature stating that coarse or clustered inclusions originate from insufficient melt cleanliness and poor deformation during rolling [27]. A considerable variation appeared among E and F specimens, where coarse inclusions predominated, indicating inadequate ladle refining and poor control of solidification, which can severely impair toughness and fatigue resistance. Such inclusions are known to crack initiation sites under cyclic loading, leading to failure [27,28].

### 3.3. Mechanical Properties

**Figure 3:**
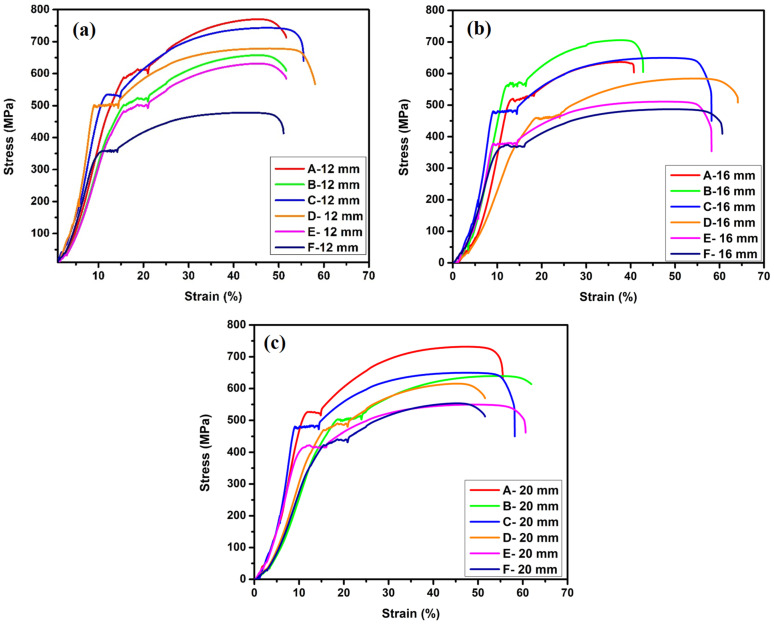
Stress–strain curve obtained from tensile testing of steel rebars from various manufacturers (A–F) investigated at three different diameters: (**a**) 12 mm, (**b**) 16 mm, and (**c**) 20 mm.

Stress–strain plots obtained from the tensile test are shown in Figure 3, and the summarized mechanical properties are presented in Table 3. Yield strength values range from 365 MPa to 580 MPa, with manufacturer A achieving the highest for 12 mm bars (580 MPa), while F consistently showed the lowest yield strength in all sizes. Similarly, ultimate tensile strength (UTS) follows a similar trend, peaking at 760 MPa for A (12 mm) and reducing to 482 MPa for the F manufacturer. The tensile to yield ratio remains within the typical range of 1.15–1.42, indicating adequate strain hardening for most samples. F shows limited improvement, indicating poor work hardening ability [29].

The variation in stress–strain behavior among 12 mm, 16 mm, and 20 mm samples is mostly attributed to diameter-dependent cooling rate during the TMT process. Smaller-sized bars cool more rapidly and experience faster heat transfer during quenching, resulting in a thicker and uniform martensite rim that increases yield and tensile strength [30]. This is consistent with the higher YS and TS achieved for the A-12 mm sample (580 MPa and 760 MPa). Conversely, bars with a diameter of 16 mm and 20 mm exhibit slower cooling rates, producing a thinner martensitic case and a wider transition zone. Consequently, the stress–strain curve for the F-20 mm sample shows lower YS and TS at 419 MPa and 538 MPa, respectively.

Microstructural investigation validates the mechanical trends for 20 mm rebars. Samples B and D, which show a relatively clear and continuous martensite rim along the case, contribute to the higher tensile strength [31]. This rim acts as a barrier to crack propagation, improving strain-hardening capability, as reported in the literature. Manufacturers C and D showed a moderate martensitic rim, resulting in acceptable elongation, 14% and 15%, respectively. But they had slightly lower tensile strength compared to A and B (Table 3). In contrast, E and F lacked a uniform rim, and in some regions, it was completely absent (Figure 1F), correlating with poor yield and tensile strength (538 and 549 MPa). Uneven distribution, large inclusions, and the absence of a martensite rim cause early failure of steel rebars [13]. These findings confirm that achieving balanced mechanical properties in rebars involves rim growth and refining inclusions to the desired morphology [32,33].

The examination of the tensile parameters against the benchmarked ASTM A615 Grade-60 standard indicates considerable performance differences amongst commercial producer batches. While samples A, B, and C consistently satisfy all mechanical properties criteria, surpassing the required thresholds for yield strength (>420 MPa) and tensile strength (>620 MPa). Samples D and E are marginally non-compliant, while sample F fails to meet the basic structural criteria completely. Conversely, all investigated batches satisfy the standard ductility limits, indicating the local variations in industrial water-quenching configurations.

**Figure 4:**
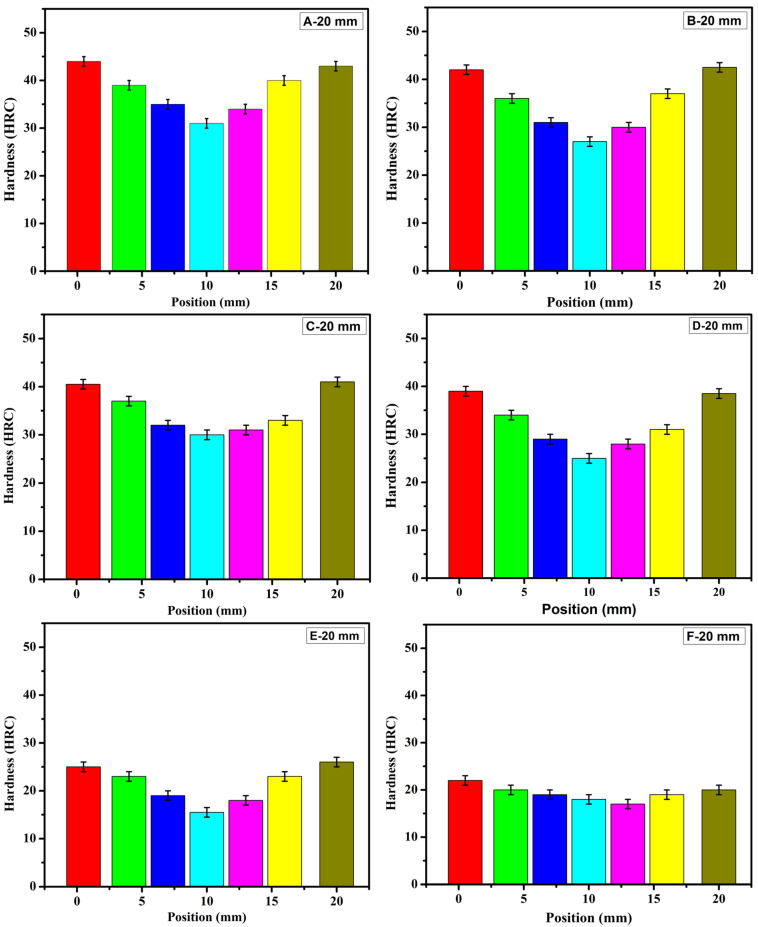
Hardness profiles of 20 mm rebars of manufacturers (A–F), displaying Rockwell C hardness (HRC) values mapped across 7 distinct positions at 1.5 mm intervals from the initial near-surface case zone to the core center region.

Figure 4 illustrates the hardness profile of 20 mm rebars of each industry. Hardness profiles show differences across cross-sections. Hardness values were mapped from the surface to the core across 7 different positions spaced at 1.5 mm intervals, revealing clear metallurgical differences across the rebar cross-sections. A and B specimens exhibit high hardness of 44 HRC and 42.5 HRC, respectively, which decreases gradually towards the core. It has been reported in the literature that high hardness increases fatigue life of structures by restricting crack propagation. Samples C and D have relatively lower hardness (40.5 HRC and 39 HRC) and at the core (29 HRC and 26 HRC), suggesting low pressure of water in the quenching system, while E and F demonstrate low hardness (26.5 and 25 HRC) with minimum case–core difference. These results agree with the microstructures obtained, which showed no martensite phase formation and a poor cooling effect. The presence of these types of features is attributed to lower tensile strength, indicating that the continuity and availability of martensite rim is essential for both mechanical strength and structural integrity. An optimized microstructure consisting of a continuous martensitic rim surrounding a core of pearlite and ferrite provides a synergistic combination of properties. The martensitic rim contributes high hardness and wear resistance, while the pearlite–ferrite core ensures toughness and ductility, reducing the risk of brittle fracture. This gradient structure allows the component to withstand surface stresses without compromising the ability to absorb impact or deformation internally. Therefore, achieving a uniform and uninterrupted martensitic rim, in conjunction with a balanced pearlite–ferrite core, is critical for enhancing both mechanical strength and structural integrity.

### 3.4. Corrosion Investigation

**Figure 5:**
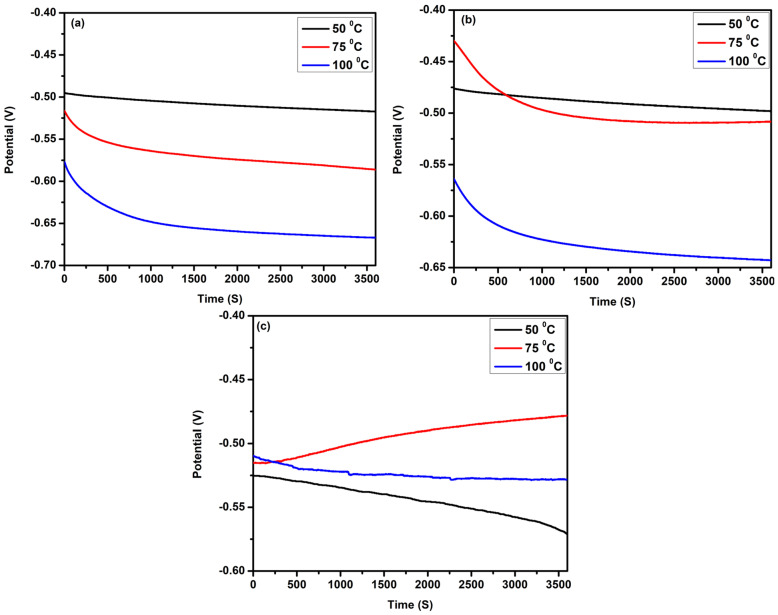
OCP curves of steel rebars immersed in seawater across three distinct temperatures: 50 °C, 75 °C, and 100 °C for (**a**) manufacturer A, (**b**) manufacturer C, and (**c**) manufacturer F.

The open-circuit potential (OCP) data for manufacturers A, C, and F exhibit distinctive electrochemical behavior affected by microstructure features under a high-temperature corrosion environment. To maintain a manageable experimental matrix while adequately evaluating the market landscape, a representative subset of rebars comprising samples A, C, and F was chosen for temperature-dependent electrochemical testing. These specific variants were selected as they encompass the high, intermediate, and low boundaries of carbon equivalents and mechanical profiles established across the broader benchmark cohort. High-temperature electrochemical measurements were executed in the natural seawater. As depicted in Figure 5a–c, manufacturer A exhibits the most stable potential (0.49 V) at 50 °C. At temperatures of 75 °C and 100 °C, initially, there is a minor drop in potential, and after 700 s the potential becomes stable. This indicates a complete martensitic rim capable of sustaining a consistent passive-like condition. Manufacturer B shows somewhat better stability at a lower temperature. Similarly, manufacturer C displays an irregular pattern at different temperatures. This type of instability may be caused by the creation of a high density of microgalvanic sites that drive much more rapid anodic and cathodic reactions in pearlite–ferrite microstructures.

**Figure 6:**
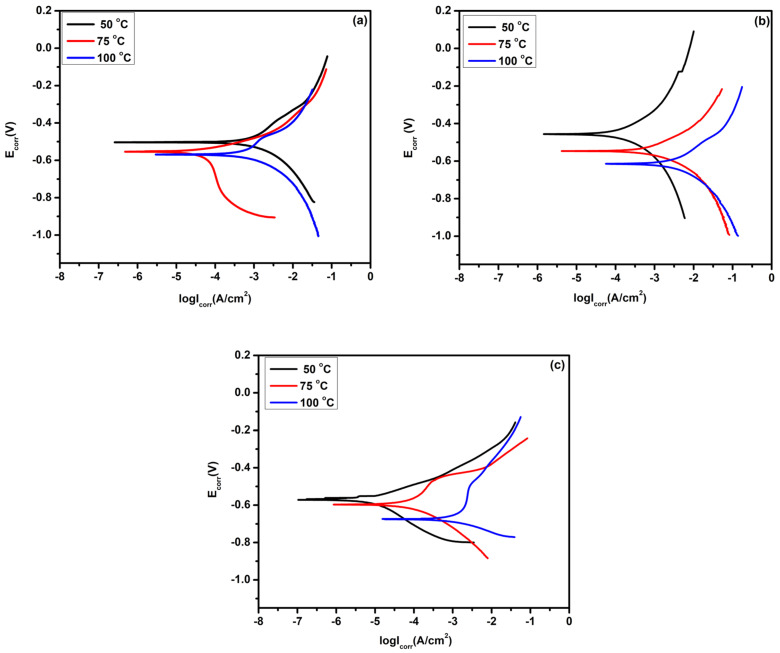
Tafel polarization plots of steel rebars immersed in seawater across three distinct temperatures 50 °C, 75 °C, and 100 °C: (**a**) manufacturer A, (**b**) manufacturer C, and (**c**) manufacturer F.

The macrophases and microstructural characteristics produced during the thermomechanical treatment (TMT) process primarily control the elevated-temperature electrochemical properties of the steel rebars in natural seawater collected from the Karachi beach area [21,34,35]. As shown in the open-circuit potential (OCP) profiles, potentiodynamic Tafel polarization plots, and extracted parameters (Figure 5 and Figure 6, and Table 4), the corrosion kinetics are heavily governed by the uniformity of the peripheral tempered martensite rim. Rebar variants featuring a clearly defined, continuous martensitic rim (such as manufacturer A) act as an effective physical barrier against aggressive marine ions. This structural shielding allows manufacturer A to maintain exceptionally low corrosion rates (*C*_R_) of 0.07 mm/y and 0.82 mm/y at 50 °C and 100 °C, respectively. This corresponds to lower corrosion current densities ($-{\log I}_{\mathrm{corr}}=4.12{\;A/\mathrm{cm}}^{2}$ at 100 °C) and a well-preserved charge transfer resistance (R_CT_ = 16.35 Ω). Conversely, as demonstrated by the dramatic rightward shift of the polarization curves in Figure 6c and data in Table 4, the absence of a martensitic rim in sample F exposes a poorly protected ferrite–pearlite surface matrix. This lack of protection causes R_CT_ to degrade to a minimal 5.93 Ω, triggering a sharp acceleration in the corrosion rate to 21.87 mm/y at 100 °C. Thermodynamically, increasing the temperature from 50 °C to 100 °C systematically drives the steady-state potentials toward more active (negative) values—with sample F dropping from 0.591 V to 0.719 V, due to accelerated anodic dissolution kinetics and diminished charge-transfer resistance across the interface [36,37]. However, a highly irregular trend is captured in sample F’s OCP profile at 75 °C (Figure 5c), where the potential undergoes a temporary noble shift. In a natural seawater matrix, this anomaly is attributed to competitive surface kinetics: at intermediate temperatures (75 °C), calcareous ions (Ca^2+^/Mg^2+^) and dissolved oxygen inherent to the natural environment react with the active ferrite matrix to temporarily precipitate a semi-protective mineral/corrosion product scale on the surface. As the temperature reaches 100 °C, this transient environmental scale undergoes rapid thermal breakdown and severe localized pitting under intense, uninhibited Cl^−^ attack.

The literature shows that fresh, untempered martensite is highly strained and packed with dislocations, which can actually accelerate local galvanic corrosion [38]. The martensitic rim produced during thermomechanical treatment (TMT) behaves differently because it undergoes self-tempering. This self-tempering relieves built-in stresses and transforms the outer layer into a tough, stable barrier against corrosion [39,40,41]. Steel cleanliness plays an equally vital role; superior manufacturers control their melt chemistry to ensure that inclusions are fine and well-dispersed, minimizing potential pitting sites. On the other hand, the coarse clustered inclusions found in poorly processed rebars create active anodic sites that speed up matrix dissolution [42,43]. This combined effect is clearly reflected in the 100 °C electrochemical data in Table 4. Sample A, which pairs an optimized tempered martensitic rim with high melt cleanliness, manages to restrict its corrosion current density to a much lower value. In contrast, sample F lacks this protective outer rim and suffers from unrefined inclusion clusters, causing its current density to drop. This represents an actual corrosion current magnitude that is nearly two orders of magnitude higher under identical thermal conditions. Ultimately, these trends underscore that having a continuous, tempered martensitic rim, alongside strict control over inclusion refinement, is essential for keeping steel rebars structurally and electrochemically stable in harsh marine conditions.

**Figure 7:**
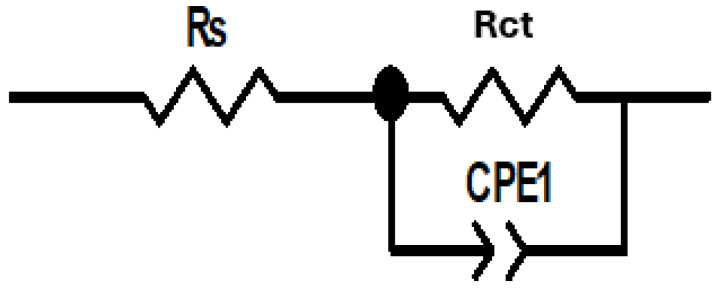
An equivalent electrical circuit corresponding to the corrosion process on the steel in seawater.

The EIS data were analyzed by fitting Nyquist plots using the Randles equivalent circuit in Z-View Software (Version 4), shown in Figure 7. This model consists of solution resistance (Rs) in series with a parallel combination of charge transfer resistance (R_ct_) and a constant phase element (CPE).

**Figure 8:**
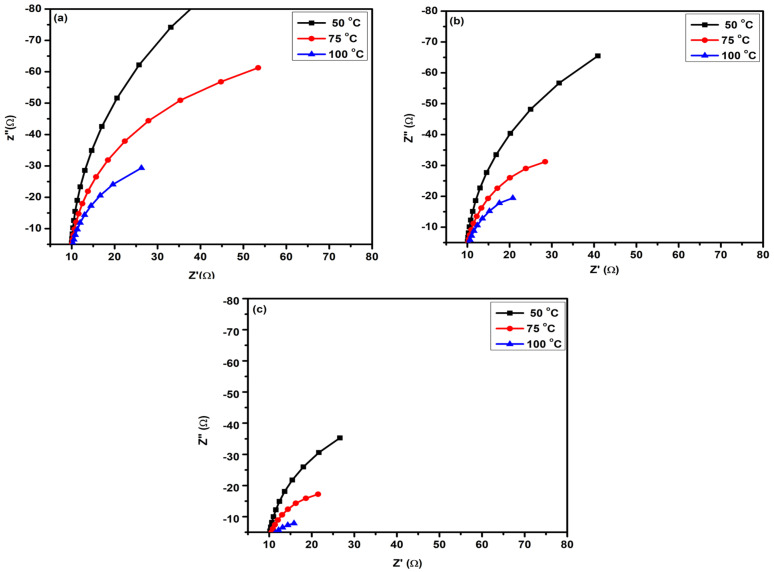
Nyquist curves of steel rebars immersed in seawater across three distinct temperatures, 50 °C, 75 °C and 100 °C: (**a**) manufacturer A, (**b**) manufacturer C, and (**c**) manufacturer F.

The EIS plots of the studied steel manufacturer display a single capacitive arc across all temperatures. This is an indication that the corrosion process is mainly controlled by a single charge transfer mechanism. As shown in Figure 8, it can be observed that the diameter of the semicircle decreased as the temperature rose from 50 °C to 100 °C. The highest arc is for sample A at 50 °C and the smallest for sample C at 100 °C. For sample A, the charge transfer resistance (R_ct_) reduces from 47.69 Ω to 16.35, and the same trend was observed for samples C and F. This reduction in impedance values is associated with thermally activated electrochemical kinetics and enhanced ion mobility in the electrolyte.

## 4. Conclusions

This study evaluated locally produced steel rebars from various local induction furnace (IF)-based manufacturers to assess both their compliance with ASTM A615 standard requirements and the underlying metallurgical mechanisms governing their behavior. Sourced variants from manufacturers A and B successfully complied with Grade 60 specifications—achieving average yield strengths above 420 MPa, tensile strengths exceeding 620 MPa, and elongations within the required range (≥9%) due to a well-defined peripheral tempered martensite rim and strong case–core contrasts that provided necessary phase-hardening. Conversely, manufacturer F failed to meet G-60 standards (yield strength < 400 MPa, tensile strength < 600 MPa) due to the complete absence of a martensitic rim, revealing uneven industrial process control. Temperature-dependent electrochemical pathways in simulated seawater (3.5 wt.% NaCl) further demonstrated that microstructural uniformity dictates corrosion kinetics; manufacturer A displayed the highest thermodynamic stability, maintaining lower corrosion rates (0.07 to 0.82 mm/y) across all temperatures, whereas manufacturers C and F exhibited accelerated degradation at elevated temperatures (75 °C to 100 °C) driven by rapid thermal breakdown of the surface oxide scale and aggressive localized Cl^−1^ ion attack. Ultimately, these findings demonstrate that relying solely on static technological standards like ASTM A615 masks critical subsurface microstructural variations that vastly alter service life. To eliminate market non-uniformity and guarantee long-term structural reliability, commercial manufacturers must move beyond simple compliance checks and actively optimize their thermomechanical treatment parameters, specifically maintaining constant quenching pressures and exact alloy chemistry, to control detrimental inclusion chemistry and prevent localized microstructural defects.

## Figures and Tables

**Table 1:** Processing and thermomechanical treatment (TMT) parameters for 20 mm steel rebars from manufacturers A–F.

IDs	Melting	Casting	Reheating	Finish Rolling	Quench Pressure	Cooling Bed
	°C	°C	°C	°C	Bar	°C
A	1543	1523	987	742	12.3	622
B	1535	1492	990	695	10.9	601
C	1543	1532	1004	706	11.6	622
D	1528	1510	969	722	10.1	618
E	1539	1497	997	728	9.8	663
F	1551	1509	978	703	9.7	643

**Table 2:** Chemical composition (wt.%) and carbon equivalent (CE) of steel rebars for different bar diameters 12 mm, 16 mm, and 20 mm sourced from various manufacturers (A–F), as determined by optical emission spectroscopy (OES).

IDs	Size (mm)	Fe	C	Si	Mn	P	S	Cu	Ni	Cr	Mo	V	N	C.E
		(wt.%)												(%)
A	12	97.929	0.265	0.289	0.805	0.041	0.043	0.284	0.075	0.246	0.012	0.004	0.007	0.476
B		97.921	0.246	0.268	0.832	0.041	0.042	0.296	0.083	0.246	0.013	0.004	0.008	0.463
C		98.076	0.225	0.258	0.770	0.041	0.039	0.284	0.073	0.209	0.013	0.004	0.008	0.422
D		98.156	0.217	0.158	0.621	0.040	0.042	0.341	0.097	0.297	0.013	0.004	0.014	0.413
E		98.033	0.224	0.164	0.710	0.040	0.047	0.362	0.103	0.290	0.014	0.005	0.008	0.435
F		98.128	0.217	0.201	0.751	0.037	0.040	0.314	0.083	0.207	0.013	0.005	0.004	0.414
A	16	97.997	0.215	0.203	0.774	0.044	0.049	0.366	0.104	0.225	0.015	0.004	0.004	0.424
B		97.92	0.229	0.250	0.844	0.037	0.035	0.298	0.093	0.264	0.019	0.005	0.006	0.453
C		98.099	0.211	0.223	0.698	0.039	0.036	0.319	0.103	0.245	0.018	0.004	0.005	0.409
D		97.953	0.205	0.257	0.761	0.043	0.043	0.333	0.094	0.286	0.013	0.005	0.007	0.421
E		98.103	0.230	0.207	0.715	0.042	0.041	0.336	0.099	0.199	0.014	0.005	0.009	0.422
F		98.087	0.219	0.222	0.782	0.042	0.044	0.278	0.088	0.215	0.013	0.003	0.007	0.420
A	20	98.099	0.234	0.206	0.775	0.033	0.039	0.283	0.081	0.222	0.014	0.003	0.011	0.435
B		98.02	0.228	0.223	0.780	0.035	0.038	0.301	0.095	0.250	0.015	0.004	0.011	0.438
C		98.105	0.229	0.220	0.750	0.034	0.034	0.288	0.082	0.234	0.013	0.004	0.007	0.429
D		97.971	0.218	0.238	0.744	0.040	0.039	0.333	0.105	0.283	0.015	0.004	0.010	0.432
E		98.007	0.210	0.231	0.755	0.049	0.047	0.360	0.107	0.202	0.016	0.004	0.012	0.411
F		98.103	0.201	0.197	0.729	0.033	0.037	0.318	0.086	0.263	0.015	0.005	0.013	0.406

**Table 3:** Summary of mechanical properties obtained from tensile testing of steel rebars across different diameters 12 mm, 16 mm and 20 mm for manufacturers A–F, and specified limits according to ASTM A615 G-60.

Sample ID	Size (mm)	Yield Strength	Tensile Strength	Tensile to Yield Ratio	Elongation
		MPa	MPa		%
ASTM A615 G-60	-	420	620	1.25 (min)	7% (12 mm and 16 mm) 9% (20 mm)
A	12	580	760	1.31	14
B		505	658	1.30	13
C		523	743	1.42	12.5
D		496	679	1.37	10.5
E		471	629	1.34	13
F		370	482	1.30	12
A	16	510	638	1.25	13
B		560	704	1.26	12.5
C		476	548	1.15	15
D		448	583	1.30	17
E		379	514	1.36	9.5
F		365	489	1.34	11
A	20	512	704	1.38	12
B		499	637	1.28	14
C		473	622	1.32	13
D		469	605	1.29	15.5
E		423	549	1.30	12
F		419	538	1.28	10.5

**Table 4:** Electrochemical parameters obtained from Tafel curves and Nyquist plots in seawater at it different temperatures (50 °C, 75 °C, and 100 °C) for manufacturers A, C, and D.

Temperature	−E_corr_	−logI_corr_	−β_a_	β_c_	C_R_	R_ct_
(°C)	(V)	(A/cm^2^)	(V/dec)	(V/dec)	mm/y	Ω
Sample A						
50	0.503	5.15	209	172	0.07	47.69
75	0.523	4.83	204	197	0.16	43.55
100	0.595	4.12	195	219	0.82	16.35
Sample C						
50	0.455	4.83	200	166	0.13	31.23
75	0.545	3.67	189	214	2.49	20.05
100	0.612	2.98	131	240	11.29	10.84
Sample F						
50	0.591	4.48	201	188	0.41	16.58
75	0.622	3.92	193	231	1.45	11.57
100	0.719	2.15	189	274	21.87	5.93

## Data Availability

The original contributions presented in this study are included in the article. Further inquiries can be directed to the corresponding authors.
